# Impact of a hospice rapid response service on preferred place of death, and costs

**DOI:** 10.1186/s12904-015-0065-4

**Published:** 2015-12-23

**Authors:** Heather Gage, Laura M. Holdsworth, Caragh Flannery, Peter Williams, Claire Butler

**Affiliations:** School of Economics, University of Surrey, Guildford, GU2 7XH England; Centre for Health Services Studies, Cornwallis Building, University of Kent, Canterbury, CT2 7NF England; Department of Mathematics, University of Surrey, Guildford, GU2 7XH England; Pilgrims Hospices in East Kent, 56 London Road, Canterbury, CT2 8JA England

**Keywords:** Preferred place of death Rapid response service Costs

## Abstract

**Background:**

Many people with a terminal illness would prefer to die at home. A new palliative rapid response service (RRS) provided by a large hospice provider in South East England was evaluated (2010) to provide evidence of impact on achieving preferred place of death and costs. The RRS was delivered by a team of trained health care assistants and available 24/7. The purpose of this study was to (i) compare the characteristics of RRS users and non-users, (ii) explore differences in the proportions of users and non-users dying in the place of their choice, (iii) monitor the whole system service utilisation of users and non-users, and compare costs.

**Methods:**

All hospice patients who died with a preferred place of death recorded during an 18 month period were included. Data (demographic, preferences for place of death) were obtained from hospice records. Dying in preferred place was modelled using stepwise logistic regression analysis. Service use data (period between referral to hospice and death) were obtained from general practitioners, community providers, hospitals, social services, hospice, and costs calculated using validated national tariffs.

**Results:**

Of 688 patients referred to the hospice when the RRS was operational, 247 (35.9 %) used it. Higher proportions of RRS users than non-users lived in their own homes with a co-resident carer (40.3 % vs. 23.7 %); more non-users lived alone or in residential care (58.8 % vs. 76.3 %). Chances of dying in the preferred place were enhanced 2.1 times by being a RRS user, compared to a non-user, and 1.5 times by having a co-resident carer, compared to living at home alone or in a care home. Total service costs did not differ between users and non-users, except when referred to hospice very close to death (users had higher costs).

**Conclusions:**

Use of the RRS was associated with increased likelihood of dying in the preferred place. The RRS is cost neutral.

**Trial registration:**

Current controlled trials ISRCTN32119670, 22 June 2012.

## Background

Many people faced with terminal illness would prefer to die at home [1–3], but less than a third do so in the United Kingdom (UK); most die in National Health Service (NHS) hospitals [2, 4, 5]. Patients are more likely to die at home if their carers receive professional support [6, 7]. The Department of Health (DH) policy guidance stresses the importance of giving people more choice over where they die and tasks NHS Trusts to provide services 24/7 to enable people to receive the care they needed to die at home [8].

Patients with life-limiting conditions are often admitted to hospital because of a crisis that could not be resolved at home, such as uncontrolled symptoms, carer fear or stress, and not having medication available [9]. Research has shown that patients who spend more time in hospital or hospice during their illness are more likely to die there [7], so keeping patients out of inpatient facilities may help improve the likelihood that patients die at home. Evidence is lacking, however, on the costs of palliative care in different settings, creating uncertainties for service commissioners [5, 8, 10–12].

Independent hospices play an important role in end-of-life care, providing inpatient, day and domiciliary care [5]. Typically, hospice services integrate with other local providers, and many different models of care exist [13]. Rapid response services (RRS) provide intense care over relatively short periods when crises arise, and work alongside regular domiciliary services that offer longer term support, to help avoid admission to hospice or hospital. Three studies in the UK [10, 14, 15] found that RRSs resulted in higher proportions of patients dying at home, compared to the national average (42 %, 41 %, 29 % vs. 21 %) [4]. However, these were small evaluations, and further research is required to provide a firm evidence base.

This paper presents the results of an evaluation of a new RRS introduced by Pilgrims Hospices in South East England in 2010 [16]. The objectives were to (i) compare the characteristics of users of the RRS with those of people that did not use it, (ii) explore differences between users and non-users in the proportions of patients dying in the place of their choice, and (iii) monitor the overall service utilisation of users and non-users, and compare costs.

## Methods

### Design

**Fig. 1:**
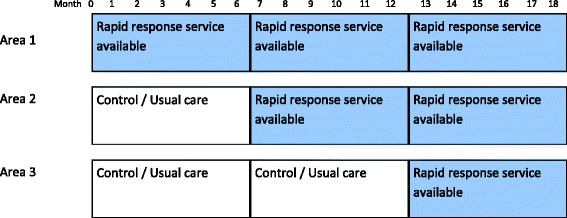
Timeline of introduction of the Rapid Response Service

Pilgrims Hospice services are delivered from three centres serving contiguous communities (total population 600,000) in the county of Kent. In each centre, the hospice provides a 16-bed inpatient ward, day hospice services and community outreach. The study followed a randomised stepped wedge design. The new RRS was rolled out sequentially to the three areas (order determined randomly using a simple probabilistic model), starting January 2010, with six months between the start of provision in each area. The study continued for six months after the RRS was introduced in the third area (total of 18 months). The time before the introduction of the RRS was deemed the control period, i.e. 0, 6 and 12 months in Areas 1, 2 and 3 respectively (Fig. 1). Once available in an area, any patients referred to the hospice in that area could access the RRS, although not all patients did. A comparison of the intervention (when RRS was provided) and control (no RRS available) conditions, and carer outcomes and experiences are reported elsewhere [17]. This paper focusses on the time when the RRS was available in each area and a comparison of the people using it with those who did not.

### RRS intervention

The RRS was developed in line with best practice and following a review of available evidence on hospice-at-home and other RRSs [13]. It is delivered by a team of experienced health care assistants (band 3), who were trained by the hospice and supported by the full hospice multidisciplinary team. The RRS serves all three areas and has access to a service co-ordinator, medical advice, and equipment carried by car. The team responds rapidly 24/7 to crises in patients’ homes (including care homes). Patients’ needs and prognosis, and family circumstances are assessed, including patient/family preferences. Hand-on care is provided in coordination with other community services. A full description of the establishment of the RRS is reported separately [18].

### Participants

All patients newly referred to any of the three centres (from any source) for any hospice service during the 18 month study period were identified from the hospice database and included, although the analysis reported in this paper only refers to patients recruited after the RRS had been introduced in the area in which they lived. Patients were ineligible if still alive at the end of the 18 month data collection period (because place of death and total services utilisation were not known), or if already registered with the hospice in Areas 2 and 3 when the RRS was introduced (because they crossed between control and intervention conditions). Amongst eligible patients, those without a recorded preferred place of death (PPD) in the hospice notes were excluded from the analysis.

### Data collection

Hospice records of individual patients were accessed retrospectively to obtain data on: age (calculated from date of birth collected at first hospice consultation); sex; residential situation (own home alone/with a carer, residential care); date referred to hospice; initial (recorded at first hospice assessment) PPD (own home, care home, hospice, hospital, other); final PPD (if subsequent changes recorded); date of death; actual place of death (APD). Number of days in the study was computed (date of death minus date referred to hospice) and grouped (<=2; 3-14, 15-30, 31-60, >60 days). Initial and final PPD were compared with APD to establish whether or not patients achieved their PPD.

### Analysis

Data were transferred to SPSS version 20 (IBM SPSS Statistics for Windows) for analysis. Excluded patients (no recorded PPD) were compared with those included with respect to age, sex, place of residence, APD. The characteristics of RRS users were compared with patients registered with the hospice in an area with a RRS but who did not access it (non-users). Comparisons (users vs. non-users) of continuous variables (age and days in study) were made using t tests; other variables were compared using chi-square tests. The factors (age, sex, area, days in study, initial PPD, residential situation) associated with achieving the PPD were explored in two stages: first using bivariate analysis and then using stepwise (backward elimination) logistic regression modelling and including RRS user/non-user as a dummy variable. Variables entered into the model were: RRS user (vs. non-user), sex (female), age, live at home alone or with carer (vs. live in care home), Area 2 or Area 3 (vs. Area 1), number of days in study (between referral to hospice and death using 1, 8, 21, 45, 75 for each of the five time groups). The five category initial PPD was recoded into 2 variables: preference for own home (Yes/No); preference for care home (Yes/No).

### Economic evaluation

The costs of the RRS were calculated on an individual patient basis from hospice records of the number of visits and time spent in patients’ homes. To assess the extent to which the RRS substituted for other forms of health and social care, service utilisation data were collected for all participants for the time that they were in the study (referral to hospice to date of death) from seven providers: general practitioners; community services; acute (hospital) services (A&E, inpatient nights, outpatient appointments, day hospital); Marie Curie home sitting; out-of-hours services (GP/nurse home visits, telephone advice, ‘walk-in’ attendances; social services received; hospice services, other than the RRS (home, outpatient, inpatient, day hospice). The number of contacts was summed within each service type. Some GPs did not return service use data, and missing items were filled using the mean values for patients with data and who were in the study for the same time period. All other service use data were complete.

Contacts with health care staff/services were converted to costs in British pounds, 2010, using validated NHS unit costs, inclusive of oncosts and overheads [19]. Discussion with the hospice finance director confirmed that it was appropriate to apply NHS rates to hospice services. Units of service use (visits, nights, hours etc.) were multiplied by the relevant unit cost to obtain the total cost per service use item per participant. Social services provided the cost of social care packages which were compared with national tariffs [19] and found to be comparable, and hence were used as supplied. Costs of individual service use items were summed within service use types, and the grand total cost was calculated.

The distributions of service utilisation and cost data were checked for normality. Since, for most items, the data were skewed (high proportions of participants with zero usage, small numbers of participants with very high usage), comparisons were conducted using medians and inter-quartile range, and Mann Whitney U tests. Utilisation and costs of users and non-users of the RRS were compared for each service use category. The analysis was conducted separately for each of the five time periods that participants were in the study because it was expected that service use and costs would correlate positively with duration.

### Ethical approval

The study received a favourable ethical opinion from the Kent Research Ethics Committee, reference 09/H1101/75. The National Information Governance Board confirmed patient consent was not needed for use of pseudo-anonymised data sets.

## Results

### Study participants

**Fig. 2:**
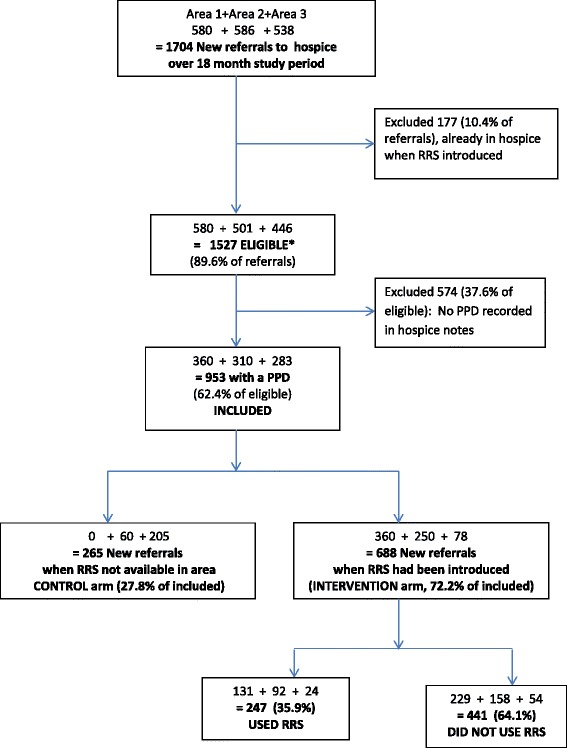
Recruitment by area. Analysis in this paper focuses on 247 users of RRS and 441 non users. * Eligible if: had died at end of study; were not already registered with hospice when RRS introduced. PPD Preferred Place of Death

The 1704 patients newly referred to the hospice during the 18 month study period were evenly distributed between the three centres (Fig. 2). Of these, 1527 (89.6 %) were eligible. Amongst the 953 for whom a PPD was known, there were 688 (72.2 %) who could potentially use the RRS because they had been referred to the hospice during the time the RRS was running in their area, and 265 who could not (because there was no RRS service available – the control group). Of the 688 who could access the RRS, 247 (35.9 %) received care from it (were RRS ‘users’) and the rest did not (‘non-users’) (Fig. 2). The proportions of users (vs. non-users) was lower in Area 3 (30.8 %) than in Areas 1 and 2 (36.6 %), but not significantly so (chi square, *p* = 0.601).

A comparison of the 953 people with a PPD and 574 excluded participants (no PPD) revealed no evidence of differences with respect to age or sex. Those with a PPD were more likely to have died at home than those without a PPD (36.9 % vs. 24.7 %) and to live alone 241 (26.2 % vs. 15.9 %) (chi sq. *p* < .001, both comparisons).

### Comparison of 247 RRS users with 441 non-users

There were no significant differences between users and non-users with respect to mean age, days in study and sex. However, users were significantly more likely than non-users to want to die at home, actually die at home, and die where they wanted. Data on residential situation were available for 586 (85.2 %) participants; higher proportions of RRS users lived in their own homes with a co-resident carer, and more non-users lived alone or in residential care. Differences were found between initial and follow-up PPD for only 12 participants (six per group) (Table 1).

**Table 1:** Comparison of characteristics of RRS users and non-users

Intervention group N = 688 (%)		Non-user (N = 441)	User (N = 247)	*P*-value
Age at death	(Mean, SD)	75.10 (12.21)	75.10 (10.22)	T test 0.985
Days in study	(Mean, SD)	69.1 (76.50)	73.1 (81.23)	T test 0.521
Area	1 Canterbury	229 (51.9)	131 (53.0)	
	2 Thanet	158 (35.8)	92 (37.2)	
	3 Ashford	54 (12.2)	24 (9.7)	
Sex	Male	245 (55.6)	143 (57.9)	χ^2^ 0.553
	Female	196 (44.4)	104 (42.1)	
Initial PPD^a^	Home	227 (51.5)	190 (76.9)	χ^2^ < 0.0005
	Care Home	47 (10.7)	2 (0.8)	
	Hospice	158 (35.8)	52 (21.1)	
	Hospital	4 (0.9)	0 (0)	
	Other	5 (1.1)	3 (1.2)	
Final PPD	Home	221 (50.1)	184 (74.5)	χ^2^ < 0.0005
	Care Home	47 (10.7)	3 (1.2)	
	Hospice	164 (37.2)	58 (23.5)	
	Hospital	4 (0.9)	0 (0)	
	Other	5 (1.1)	2 (0.8)	
APD^a^	Home	114 (26.3)	156 (63.2)	χ^2^ < 0.0005
N = 434 non-users	Care Home	65 (15.0)	11 (4.5)	
	Hospice	200 (46.1)	61 (24.7)	
	Hospital	55 (12.7)	19 (7.7)	
Residential situation	At Home not alone	218 (58.8)	164 (76.3)	χ^2^ < 0.0005
N = 371 non-users	At home alone	103 (27.8)	48 (22.3)	
N = 215 users	Residential care	50 (13.5)	3 (1.4)	
Achieving PPD (using initial PPD)	Yes	257 (59.2)	171 (69.2)	χ^2^ 0.009
N = 434 non-users	No	177 (40.8)	76 (30.8)	

*APD* Actual Place of Death

*PPD* Preferred Place of Death

^a^Each care home resident whose PPD (n = 47) and/or APD (n = 31) was recorded as ‘home’ in the hospice database was individually investigated to assess whether this referred to the ‘care home’ or to their own independent accommodation so that it could be appropriately coded. Following enquiries all APD, and, except for four PPD, were found to be ‘care home’

Comparing characteristics of users and non-users (taken together) across the three areas, no significant differences were found in age, gender, APD or achieving PPD, but stating a preference to die at home (vs. any other location) was significantly higher in Area 1 than in Areas 2 or 3 (65.0 % vs. 56.0 %, 55.1 %, *p* = .016 for initial PPD, 62.8 % vs. 56.4 %, 48.7 %, *p* = .004 for final PPD).

### Died where wanted

The APD was not known for 7 non-users. Of the remaining 681 participants, 171 (69.2 % of 247 RRS users) and 257 (59.2 % of 434 RRS non-users) died in the preferred place they initially stated (*p* = 0.009). Of 190 users of the RRS wanting to die at home, 141 (74.2 %) achieved that, compared to 96 of 223 (43.0 %) non-users (*p* <0.0005) (Table 2).

**Table 2:** Comparison of actual and preferred place of death

Achieved PPD shown on diagonal		APD								
		Home		Care Home		Hospice		Hospital		Total
Group	Initial PPD	N	%	N	%	N	%	N	%	N
Non user	Home	96	43.0	9	4.0	79	35.4	39	17.5	223
	Care Home	1	2.1	43	91.5	1	2.1	2	4.3	47
	Hospice	15	9.7	12	7.7	117	75.5	11	7.1	155
	Hospital	1	25.0	0	0	2	50.0	1	25.0	4
	Other	1	20.0	1	20.0	1	20.0	2	40.0	5
	Total	114	26.3	65	15.0	200	46.1	55	12.7	434
User	Home	141	74.2	2	1.1	33	17.3	14	7.3	190
	Care Home	0	0	2	100.0	0	0	0	0	2
	Hospice	14	26.9	5	9.6	28	53.8	5	9.6	52
	Other	1	33.3	2	66.7	0	0	0	0	3
	Total	156	63.2	11	4.5	61	24.7	19	7.7	247

Bivariate analysis showed that the proportion who achieved their PPD varied depending on PPD, being highest for people wanting to die in the hospice, or a care home (most of whom were already resident in a care home). Being in the study for a longer length of time (more days between referral to the hospice and death) reduced the chances of dying in the initial PPD. Age and area of residence were not significantly associated with achieving PPD (Table 3).

**Table 3:** Characteristics of those who achieved initial preferred place of death

		Achieved PPD - NO (N = 253)		Achieved PPD – YES (N = 428)		Significant difference (p)
		N	%	N	%	
Area	1	120	33.7	236	66.3	
	2	100	40.5	147	59.5	χ^2^ 0.144
	3	33	42.3	45	57.7	
Gender	Female	100	33.7	197	66.3	χ^2^ 0.098
	Male	153	39.8	231	60.2	
Days in study	0-2	4	10.0	36	90.0	
	3-14	30	25.4	88	74.8	χ^2^ < 0.0005
	15-30	36	30.5	82	69.5	
	31-60	54	37.2	91	62.8	
	>60	129	49.6	131	50.4	
Initial PPD	Own home	176	42.6	237	57.4	
	Care home	4	8.2	45	91.8	χ^2^ < 0.0005
	Hospice	62	30.0	145	70.0	
	Hospital	3	75.0	1	25.0	
	Other	8	100.0	0		
		Achieved PPD - NO (N = 225)		Achieved PPD – YES (N = 354)		
Residential situation	Own home not alone	146	38.7	231	63.1	
	Own home alone	69	46.3	80	53.7	χ^2^ 0.002
	Care home	10	18.9	43	81.1	

Regression modelling showed that being a RRS user enhanced the chances of dying where initially wanted 2.1 times compared to being a RRS non-user. A person living at home with a co-resident carer (i.e. not alone) was 1.5 times more likely to achieve their PPD compared to anyone living at home alone or in a care home. Stating an initial PPD as a care home afforded a 7.7 times greater chance of achieving their PPD compared to those with a PPD in any other location. Stating an initial PPD as own home afforded a 0.55 times less chance of achieving PPD than stating a PPD in any other location (Table 4).

**Table 4:** Stepwise (backward elimination) logistic regression modelling of achieving PPD (Yes/No)

Variable	Odds Ratio (OR)	*p*	95 % CI for OR	
			Lower	Upper
Live at home with carer	1.505	.050	1.001	2.263
Area 3	0.542	.036	0.306	0.959
RRS user (vs. non-user)	2.099	<.0005	1.430	3.081
Days in study (between referral to hospice and death)	0.983	<.0005	0.977	0.989
Initial PPD is home	0.548	.005	0.362	0.830
Initial PPD is care home	7.708	<.0005	2.531	23.470
Constant	2.894	<.0005

**Table 5:** Total service use: comparison of RRS users (N = 247) and non-users (N = 441), by days in the study

		Days in study (time between referral to hospice and death)									
Service type		0-2 days		3-14 days		15-30 days		31-60 days		>60 days	
	Study Group	Non-User	User	Non-User	User	Non-User	User	Non-User	User	Non-User	User
	Number in Group	17	7	52	28	37	30	58	36	103	58
All GP/primary contacts^a^	No (%) with > =1 contact	5 (29.4)	4 (57.1)	22 (42.3)	19 (67.9)	25 (67.6)	27 (90.0)	46 (79.3)	33 (91.7)	94 (91.3)	56 (96.6)
	Median contacts, IQR	0,0-1	1,0-1	0,0-1	1,0-3	1,0-2	2,0.1-2	2,0-4	2.5,0.7-4	4,1-8	6,1.9-8.5
	MWU	0.285		0.011		0.078		0.539		0.031	
	Number in Group	31	9	76	42	66	52	100	47	168	97
All Community contacts^b^	No (%) with > =1 contact	16 (51.6)	8 (88.9)	36 (47.4)	34 (81.0)	39 (59.1)	45 (86.5)	83 (83.0)	43 (91.5)	132 (78.6)	93 (95.9)
	Median contacts, IQR	1,0-4	7,0-8	0,0-5	11.5,0-17	2.6,0-7.5	13.5,0-21.25	7.5,0-15.75	15,0.80-34	12,0-30.5	26,5-43
	MWU	0.024		0.000		0.000		0.004		0.000	
All Acute contacts^c^	No (%) with > =1 contact	1 (3.2)	0 (0)	19 (25.0)	3 (7.1)	30 (45.5)	19 (36.5)	64 (64.0)	25 (53.2)	135 (80.4)	82 (84.5)
	Median contacts, IQR	0,0-0	0,0-0	0,0-0.75	0,0-0	0,0-2	0,0-1.75	1,0-7.75	1,0-5	6,0-15	4,0-12
	MWU	0.590		0.014		0.426		0.369		0.231	
All Marie Curie visits^d^	No (%) with > =1 contact	1 (3.2)	0 (0)	0 (0)	7 (16.7)	0 (0)	9 (17.3)	2 (2.0)	5 (10.6)	3 (1.8)	12 (12.4)
	Median visits, IQR	0,0-0	0,0-0	0,0-0	0,0-0	0,0-0	0,0-0	0,0-0	0,0-0	0,0-0	0,0-0
	MWU	0.590		0.000		0.000		0.022		0.000	
All Out-of-hours contacts^e^	No (%) with > =1 contact	2 (6.5)	2 (22.2)	8 (10.5)	16 (38.1)	11 (16.7)	18 (34.6)	20 (20.0)	17 (36.2)	43 (25.6)	46 (47.4)
	Median contacts, IQR	0,0-0	0,0-0	0,0-0	0,0-1	0,0-0	0,0-1.75	0,0-0	0,0-1	0,0-1	0,0-2
	MWU	0.170		0.000		0.016		0.034		0.000	
Hospice Contacts^f^, not RRS	No (%) with > =1 contact	31 (100)	9 (100)	76 (100)	42 (100)	66 (100)	52 (100)	100 (100)	47 (100)	168 (100)	97 (100)
	Median contacts, IQR	1,1-3	1,1-1.5	4,1-7.75	3,1-4	10,1.7-16.2	3.5,2-6	4.5,2-13.5	6,2-13	11,2-28	13,4-26.5
	MWU	0.170		0.404		0.000		0.161		0.302	
Social services^g^	No (%) > =1 service	1 (3.2)	1 (11.1)	8 (10.7)	3 (7.1)	5 (7.6)	9 (17.3)	22 (22.0)	6 (12.8)	24 (14.3)	21 (81.6)
	MWU	0.345		0.536		0.104		0.158		0.167	
Hospice RRS	No (%) used RRS	0	9 (100)	0	42 (100)	0	52 (100)	0	47 (100)	0	97 (100)
	Median visits, IQR		4,1-8		11,2-23		9.50,1-27		12,1-33		14,1.8-33.5
	Median hours, IQR		8,4-9.2		15.1,4-28.4		10,2-31.5		12.7,2.9-50.4		18,2-47.9

Notes:

^a^Sum of patient visits to surgery to see GP or practice nurse, and home visits by GP

^b^Sum of visits and telephone calls to patient by community nurse, long term condition team, intermediate care teams, community matron

^c^Sum of visits to hospital A&E, inpatient nights, outpatient appointments, day hospital visits

^d^Number of Marie Curie health care assistants or registered nurse visits; each lasted 8 h (overnight sitting)

^e^Sum of out of hours home visits by GP or nurse, telephone advice by GP, ‘walk-in’ attendances, and ambulance responses

^f^All participants had been referred to the hospice. Hospice services include: home or outpatient contacts with hospice nurses, doctors, allied health professionals, social worker, chaplain; inpatient stays; day hospice attendances for complementary therapies

^g^Number of social services received (e.g. domiciliary help, meals)

*MWU* Mann Whitney *U* test

IQR is displayed as the explicit 25 % - 75 % values

### Service use and costs

Users of the RRS had higher use of GP, community, Marie Curie and out-of-hours services than RRS non-users; the difference was significant for some time periods and services. Non-users tended to have higher use of acute hospital services (significant 3 – 14 day period) and hospice services other than the RRS (significant 15-30 day period) than the RRS users (Table 5).

The pattern of costs for users and non-users reflects service utilisation. The large cost items are primary, community, hospice and hospital inpatient stays. As expected, costs increase as duration of time in the study rises. However, the RRS is a crisis-driven, time-limited service, so the median number of visits to people with different times in the study was similar (overall median: 11 visits; cost £425). There was no significant difference in the total service costs of users and non-users for any time period, except, amongst those referred to the hospice within 2 days of death, when RRS users had significantly higher overall cost of services than non-users due to the RRS input and other community care costs (Table 6).

**Table 6:** Costs of services received £2010^b^ : Comparison of RRS users (N = 247) and non-users (N = 441), by days in the study

Median (inter- quartile range, 75^th^ – 25^th^),	Days in study (time between referral to hospice and death)									
	0-2 days		3-14 days		15-30 days		31-60 days		>60 days	
Study group	Non-user	User	Non-user	User	Non-user	User	Non-user	User	Non-user	User
Number in group	31	9	76	42	66	52	100	47	168	97
All GP/primarycontacts	65 (65)	65 (120)	120 (123)	123 (117)^a^	189 (69)	189 (48)	339 (217)	339 (240)	525 (273)	525 (218)^a^
All community contacts	27 (108)	189 (135)^a^	0 (133)	302 (375)^a^	63 (200)	364 (390)^a^	209 (378)	378 (814)^a^	331 (773)	737 (777)^a^
All acute (hospital) contacts	0 (0)	0 (0)	0 (54)^a^	0 (0)	0 (274)	0 (137)	93 (2718)	72 (1700)	1064 (4025)	900 (2943)
All Marie Curie visits	0 (0)	0 (0)	0 (0)	0 (0)^a^	0 (0)	0 (0)^a^	0 (0)	0 (0)^a^	0 (0)	0 (0)^a^
All Out-of-hours contacts	0 (0)	0 (0)	0 (0)	0 (41)^a^	0 (0)	0 (81)^a^	0 (0)	0 (41)^a^	0 (33)	0 (122)^a^
All hospice contacts, not RRS	90 (324)	90 (45)	504 (2056)	241 (365)	2512(5056)^a^	274 (511)	360 (1889)	605 (2919)	1694 (8016)	1172 (5707)
All social services	0 (0)	0 (0)	0 (0)	0 (0)	0 (0)	0 (0)^a^	0 (0)	0 (0)	0 (0)	0 (0)
Hospice RRS visits	0 (0)	160 (240)^a^	0 (0)	440 (721)^a^	0 (0)	380 (951)^a^	0 (0)	480 (1161)^a^	0 (0)	560 (1181)^a^
Total of 8 services	367 (262)	690 (291)^a^	1372 (2102)	1548 (1278)	3790 (4409)	2204 (2523)	3809 (6859)	5110 (5461)	7298(11327)	7324 (7951)

^a^Mann Whitney *U* test, significantly higher than the other group, *p* < 0.05

^b^Unit costs of health care, inclusive of oncosts and overheads, applied to service utilisation [19]. GP/Primary: GP/nurse surgery consultations £36/£7.75, GP home visits £120. Community: home visit by nurse/long term conditicns or intermediate care team £27/£31.40, telephone call by long term conditions or intermediate care team £10.00. Acute: A&E £114, outpatient consultation £72, day hospital £194, inpatient night £425. Marie Curie health care assistant/nurse visit: £32/£64. Out-of hours: GP/home visit £180 / £40.50, GP telephone advice £33, walk-in clinic £37, ambulance £90. Hospice costs (based on NHS costs following discussion with hospice finance director), inclusive of oncosts and overheads: home visits by consultant/associate director/nurse/physician/associate practitioner/social worker or chaplain/physiotherapist or occupational therapist: £355/£132/£90/£324/£40/£172/£225/£58; outpatient consultations with consultant/nurse/physician/associate practitioner/social worker: £213.40/£28/£140/£5.25/£106.50; inpatient days: £425; day hospice: £194; counselling: £53.25; breathlessness therapy: £38; complementary therapies (varied): £25.75; RRS: £40 per visit (two visits counted when two HCAs attended)

## Discussion

The study was based on people referred to a large hospice provider and focussed on a comparison of users and non-users of a new RRS that was rolled out in phases across three areas. When the RRS was available in an area, some 36 % of hospice clients accessed it. Users were more likely than non-users to live in their own homes with a co-resident carer and have expressed a wish to die at home. Overall there was no difference in the whole system costs of RRS users and non-users, except RRS users referred to the hospice within two days of death incurred significantly higher total costs than non-users in the study for the same period of time. Hospice patients who did not use the RRS relied on all the other hospice services more than RRS users, but, consistent with some other evidence [20], RRS users had higher utilisation of other health services than non-users.

Amongst the 953 patients for whom a PPD was known, there was no significant difference between the intervention and control arms of the study in the proportions who died in their preferred place (428 of 688, 62.8 % in the intervention group, 164 of 265 in the control group, chi squared *p* = .724) [17]. However, within the intervention group, significantly higher proportions of RRS users than non-users achieved their PPD (69 % vs. 59 %). In particular, people wanting a home death were more likely to achieve it if they had access to the RRS (74 % users vs. 43 % non-users). Being a RRS user more than doubled a participant’s chances of dying where they wanted. Other studies set in the UK have also found that RRS results in higher proportions of patients dying at home [10, 14, 15]. This might reflect selection bias, i.e. only people where health professionals perceive the RRS could make a difference are offered access to it.

Having a co-resident carer also increased the chances (one and half times) of dying in the preferred location, reflecting the well-recognised problems of facilitating the wish to die at home of people who live alone [21]. In supporting carers to minimise the adverse effects of their role [22], the RRS may prevent carer breakdown and unplanned use of inpatient services. The group most likely to die where they wanted was care home residents, most of whom were deemed to have wanted to die in their care home, and achieved that wish. It should be noted, however, that ambiguities in the hospice data that recorded the wishes of some care home residents as being to die at ‘home’ were interpreted by the researchers (after case-by-case investigation) as meaning ‘care home’.

Patients who were referred to the hospice further from death, and who wanted to die at home, were less likely to achieve that than those referred to the hospice closer to death and those who wanted to die somewhere other than their own home. The analysis was based on initial PPD as recorded in the hospice notes, and lower proportions dying where they wanted amongst people in the study longer could have arisen because changes in wishes were not recorded. It is known that preferences change, even in the last hours [23], with lower proportions wanting to die at home as death approaches [5]. Wishes are influenced by unforeseen experiences, the views and health of family carers and by the care process itself, e.g. the availability of technology at home. The extent to which changes in patients’ wishes as death approached were captured in this study is not known. Analysis revealed differences between the initial and final wishes in only 12 participants, but recording of changes of preference was not reliable, and it is possible that changes to the initial PPD were overwritten in notes. Although meeting individual’s PPD is a suggested national outcome for palliative services [8, 24], its use in practice, and in research, presents challenges and findings based on it, including statistics on achieving PPD, should be interpreted with caution.

The RRS was delivered by the same team in all three areas and data were aggregated for analysis. Prior to the service being rolled out in each area, additional HCAs were recruited. The RRS worked as a team, and each HCA visited patients across all three areas [18]. Although patients did not differ between areas in age, sex, residential situation, and length of time in the study, higher proportions of patients in Area 1 expressed a wish to die at home, and participants in Area 3 had a lower chance of dying in their preferred place, after controlling for all other factors. Reasons underlying these differences are not known, but may reflect differing socio-economic profiles of the areas.

The study meets a well-recognised need for information on end-of-life costs to inform service commissioning [5, 8, 10–12] and provides unique information on whole system utilisation of services in the days/weeks leading up to death. The data were meticulously collected from hospice records and directly from providers, and the findings confirm the high resource use of palliative care [25]. However, unlike some other studies [10, 26–29], provision of the RRS was not associated with lower overall costs, and this may reflect the particular context in which this RRS was delivered and the patient group it covered. Decisions about who was offered the RRS were usually made by hospice clinical staff based on whether immediate end-of-life care was needed (prognosis less than 72 h) or for crisis intervention (sudden deterioration or carer breakdown). People receiving the RRS were disproportionately those with co-resident carers, had expressed a wish to die at home and had high utilisation of other services. Some other specialist palliative care services have different referral criteria, for example, only including people wanting to die at home [28]. From a commissioning perspective, it is interesting to note that non-users of the RRS tended to have higher utilisation of hospital and other hospice services, and further work is needed to explore this trend.

Methodological and ethical challenges confront research in the palliative care arena, and specific problems are associated with randomisation, and measuring quality of life and outcomes [30–32]. Particular difficulties exist in calculating palliative care costs [8, 10, 11] due to problems defining when end-of-life starts [33], and the fragmentation of service use data across providers [26, 34]. This study took a pragmatic approach and utilised the opportunity provided by the introduction of a new RRS (a natural experiment) to evaluate its impact on achieving PPD and costs. Performing a retrospective power calculation, based on detecting a 10 % difference between users and non-users of the RRS in achieving PPD, the sample sizes (247 vs. 434) provided good power (74.2 % for a 2-sided test).

However, it has several limitations. It only includes people referred to a hospice, and this group may not be representative of all people receiving community palliative care. People with no recorded PPD were excluded, and this group was less likely to have died at home and live alone than those with a PPD, so the sample analysed was not entirely typical of the whole hospice population. Service use data and costs were analysed in five time periods which were determined *post hoc*. Experimentation suggested that a finer (weekly) division (13 time periods) was impractical because the group sizes were too small for meaningful analysis. The five groups identified provided approximately even numbers of participants (except the open-ended longest period), but different groupings could have affected the findings. Some GPs did not provide service use data (affected 37 % of participants) and use of mean imputation may have introduced inaccuracies. The costs of the RRS may be underestimated since they were based on visits lasting one hour, and sometimes staff stayed longer (e.g. night sitting). Travel costs (averaged £12 per visit) were not included since they varied with the location of the patient’s home. The burden of a home death on family carers was not calculated although evidence shows this exceeds the cost of formal services [35]. No measure of quality of care was included although interviews with a sample of carers in the study identified that care providers have an important influence on a ‘good death’ [36].

## Conclusions

The benefits of an integrated care system to address the multiple and diverse needs of people at the end of life are recognised [37], and a RRS plays an important role within that system by increasing choice and reducing the fragmentation of services that is a source of distress to patient and carers [5, 8, 11, 38–40]. Amongst patients referred to the hospice in this study, those with a co-resident family carer and who had expressed a wish to die at home were most likely to receive care from the RRS. The provision of the RRS that worked in cooperation with other local services did not affect the whole system costs of providing palliative care. Analysis of users compared to non-users produced evidence that the RRS supports people to die where they want, especially if that is in their own home. But there is a high likelihood of bias in the selection of patients who receive access to the RRS. Supply side factors, however, also affect outcomes, and resource constraints may have restricted access to the service and affected the proportions dying in their preferred place. Since the service was observed to be cost neutral, a case exists for widening access to it.

## Abbreviations

A&E: Accident and Emergency Department
APD: Actual Place of Death
DH: Department of Health
GP: General Practitioner
HCA: Health Care Assistant
NHS: National Health Service
PPD: Preferred Place of Death
RRS: Rapid Response Service
